# A network analysis of the relationship between perceived social support, emotion regulation, and job burnout among elementary and middle school teachers

**DOI:** 10.3389/fpsyg.2025.1704862

**Published:** 2025-10-31

**Authors:** Yueyao Li, Yuyang Nie, Zhaoming Ma, Cong Liu, Yuxian Cao, Guofeng Qu, Lijia Hou, Kangli Du, MingMing Guo, Tianci Wang, Xilai Zhu

**Affiliations:** ^1^College of Physical Education and Sports, Beijing Normal University, Beijing, China; ^2^College of Education for the Future, Beijing Normal University, Zhuhai, China; ^3^Henan Sports, Gymnastics and Ice and Snow Sports Center, Zhengzhou, Henan, China; ^4^College of Sports, Ningxia University, Yinchuan, China; ^5^Zhuhai Middle School Affiliated to Sun Yat-sen University, Zhuhai, China

**Keywords:** elementary and middle school teachers, perceived social support, emotion regulation, job burnout, network analysis

## Abstract

**Background:**

Job burnout is a common mental health problem among primary and secondary school teacher populations, and navigating perceived social support and emotion regulation are recognized as important protective factors. The aim of this study was to explore the relationship between perceived social support, emotion regulation, and job burnout among elementary and secondary school teachers and to provide an in-depth analysis using network analysis and mediation modeling.

**Methods:**

A questionnaire was administered to 351 elementary and middle school teachers using the perceived social support Scale, Emotion Regulation Scale and Job Burnout Scale. Network analysis was used to depict the network of associations between the variables, and mediation analysis was used to test the mediating role of emotion regulation between perceived social support and Job Burnout.

**Results:**

The results of the network analysis revealed that the connections within variables were stronger than those across variables. The core associations between different dimensions included: “family support” and “emotional exhaustion,” “family support” and “depersonalization,” as well as “cognitive reappraisal” and “reduced personal accomplishment.” Family support exhibited the highest Bridging Expected Influence (BEI), is the core hub of the network. The mediation model confirmed the mediating role of emotion regulation, indicating that perceived social support indirectly affects job burnout through emotion regulation. In addition, there were no significant differences in network characteristics between genders or across school stages (elementary vs. middle school).

**Conclusion:**

Elementary and middle school teachers’ perceptions of perceived social support, emotion regulation, and job burnout constitute a dynamic network of associations, with family support and cognitive reappraisal as the core nodes, and emotion regulation mediating the role of perceived social support in alleviating job burnout. The study provides a theoretical basis and intervention target for improving teachers’ mental health by strengthening family support and cognitive reappraisal.

## 1 Introduction

In recent years, people’s attention to mental health is increasing, and the profession of teaching is highly regarded for its important contribution to the development of society, and its mental health status is especially critical. This profession not only requires unswerving dedication, but also continuous emotional investment and strong mental toughness (Mir Jalalli, 2024; Nwoko et al., 2024). For primary and secondary school teachers in particular, increasing educational pressures – including frequent curriculum reforms, heavy administrative workloads, and complex demands on student management (especially “problem students”)—have made job burnout a common and pressing problem (Wong et al., 2017; Karimi and Adam, 2023). This state of burnout not only erodes the physical and mental health of teachers, but also directly reduces the quality of teaching, ultimately damaging students’ learning outcomes (Oh and Wolf, 2023). Compounding these challenges is that teachers often grapple with the tension between traditional educational ideals and societal changing expectations of their roles; Therefore, balancing professional requirements with personal life needs to maintain mental health and professional identity has become a key practical dilemma faced by contemporary educators (Moiseeva, 2024).

Teacher job burnout is closely linked to job satisfaction. Importantly, interpersonal relationships in the workplace (including those with students, colleagues, and leaders)significantly influence teachers’ emotional well-being (Lata and Sharma, 2021). perceived social support, as an important buffer mechanism, plays an irreplaceable role in resisting work pressure and dissipating job burnout. When teachers can truly feel the understanding and tolerance from their families, the listening and encouragement from their friends, and the cooperation from their colleagues, their inner anti-stress defense will become stronger, which will enhance life satisfaction and significantly reduce Job Burnout (Ju et al., 2015). Previous research has shown that work-related relationships characterized by a lack of support and trust increase the risk of job burnout (Frögéli et al., 2019). In contrast, when these work-related relationships exhibit positive qualities and are accompanied by high levels of social support, teachers are not only able to resolve disagreements encountered at work more effectively, but are also more likely to experience higher levels of work engagement and professional well-being (Kinman et al., 2011). Support from supervisors may play a more significant role in reducing Job Burnout than support from coworkers (Zeng et al., 2024).

Teachers’ emotion regulation skills play an important role in coping with job stress and preventing Job Burnout (Yin et al., 2016). Emotion regulation refers to the process by which an individual changes his or her emotional state through various ways. Effective emotion regulation strategies can help teachers better manage their emotions and reduce the accumulation of negative emotions, thus reducing the risk of job burnout (Bing et al., 2022). Teachers are often under intense work pressure, dealing with a large number of teacher-student interactions, coping with a variety of teaching tasks, and at the same time taking care of their students’ personal development and emotional needs (López-Pérez et al., 2024). This special professional environment makes emotion regulation one of the core professional qualities required of teachers. Teachers with strong emotion regulation skills are often able to adapt to changes in the working environment more flexibly and always maintain a positive professional mindset, so that they can walk more steadily and farther in their education career (Verešová and Malá, 2012). In a study of job burnout among coaches (Su, 2012), it was found that cognitive reappraisal had a negative predictive effect on Job burnout, and expression inhibition had a positive predictive effect on job burnout. Since both coaching and teacher involve frequent emotional labor and interpersonal interaction, findings from research on coaches regarding the relationship between emotion regulation strategies and job burnout can provide theoretical reference and intervention insights for emotion management among teachers.

Previous studies have indicated that perceived social support and emotion regulation are key factors influencing teacher Job Burnout (Fiorilli et al., 2016; Mulyani et al., 2021). However, complex interactive mechanisms may exist among these variables. For instance, perceived social support may indirectly mitigate job burnout by facilitating teachers’ adoption of adaptive emotion regulation strategies (Fiorilli et al., 2016). It is worth noting that the strength of the relationships among these variables may vary across professional contexts; for example, a review study targeting nurses reported only a weak correlation between job burnout and perceived social support (Zeng et al., 2022). The theoretical framework that unifies these observations is resource conservation (COR) theory, which assumes that individuals strive to acquire, retain, and protect valuable resources (e.g., emotional energy, social relationships, self-efficacy). Burnout occurs when resources are depleted faster than they can be replenished, or when resource loss is threatened. Traditional statistical methods, such as regression analysis and structural equation modeling, exhibit limitations in capturing such complex interactions and fail to reveal the dynamic associative structure among variables. As an emerging methodology, network analysis can visually represent interactive patterns among multiple variables within a system, thereby offering a novel research perspective for deepening the understanding of the intricate relationships among perceived social support, emotion regulation, and job burnout.

Network analysis constructs a network by representing research objects as nodes and relationships between them as edges. In this study, a teacher job burnout network model was constructed by treating three scales—perceived social support, emotion regulation, and job burnout—as distinct communities, with the subdimensions of each scale serving as nodes and the correlations between these nodes forming the edges. By analyzing this network model, it is possible to identify the perceived social support and emotion regulation factors that have the greatest impact on job burnout, as well as the interaction relationship between them. At the same time, mediation analysis will be applied with a view to revealing more specific underlying mechanisms of action between the variables, thus contributing to the theoretical link between perceived social support, emotion regulation and job burnout. By constructing and analyzing the network model of teacher Job Burnout and the role of mediation, this study will deeply reveal the complex relationship between these factors and provide a scientific basis for formulating effective interventions to enhance teachers’ occupational well-being and work efficiency. The study will also help educational administrators to better understand teachers’ needs so that they can formulate more effective support policies to promote teachers’ professional development and psychological health.

## 2 Methods

### 2.1 Participants

The study adopted a cross-sectional design and collected data from teachers in two public primary and secondary schools in Handan City, Hebei Province from May to July 2025, and conducted all procedures in compliance with the Declaration of Helsinki — Ethical Principles for Medical Research Involving Human Subjects, along with other applicable laws, regulations, and ethical guidelines. Prior to the survey, all participants received formal training to ensure the integrity of the study. The STROBE checklist for cross-sectional studies was followed and a questionnaire containing basic information about the subjects, perceived social support, emotion regulation and Job Burnout was created. After confirming the consent of the teachers, a link was given to them for completion.386 participants completed the questionnaire, to ensure data quality, each questionnaire was carefully reviewed after collection, and those that were incomplete, exhibited irregular patterns, or contained illogical responses were excluded, a total of 35 questionnaires were excluded, giving a validity rate of 90.93%. There were 107 (30.5%) male teachers and 244 (69.5%) female teachers; A total of 75 teachers were unmarried and had no children (21.4%), 52 were married but had no children (14.8%), and 224 were married and had children (63.8%); 178 (50.71%) elementary school teachers and 173 (49.29%) secondary school teachers, A total of 69 teachers had less than 3 years of work experience (19.6%), 88 had 3–5 years of experience (25.1%), and 74 had 5–10 years of experience (21.1%), with more than 10 years of employment 120 (34.2%), and the average age of the participants was 37.83 ± 8.061 years.

### 2.2 Instruments

#### 2.2.1 Perceived social support (PSSS)

The Perceived Social Support Scale (PSSS) (Zimet et al., 1988) was used in this study to measure teachers’ perceived level of social support. The scale consists of 12 items across three dimensions: “Family Support,” “Friend Support,” and “Other Support.”, and all entries are scored on a Likert 7-point scale (1 = “strongly disagree,” 7 = “strongly agree”), with a total score ranging from 12–84, with a score of Higher scores indicate higher levels of perceived support. The Cronbach’s *α* value for this scale in this study was 0.943.

#### 2.2.2 Emotion regulation (ERQ)

The Chinese version of the emotion regulation questionnaire (ERQ) (Wen et al., 2023) compiled by Gross and translated by Wang Li et al. was used in this study. The questionnaire comprises two main dimensions: cognitive reappraisal and expressive suppression. The cognitive reappraisal subscale consists of 6 items, while the expressive suppression subscale includes 4 items. Responses are recorded on a 7-point Likert scale (ranging from 1 = “strongly disagree” to 7 = “strongly agree”). Higher scores indicate a greater tendency to employ the corresponding emotion regulation strategy in daily life. In this study, the overall Cronbach’s alpha for the questionnaire was 0.899.

#### 2.2.3 Job burnout

In this study, the job burnout scale compiled by Professor Li Yongxin was used to evaluate the job burnout of primary and secondary school teachers (Yongxin and Mingzheng., 2005). The scale is tailored to the local educational context, so it is more suitable for measuring job burnout among primary and secondary school teachers in China, the scale includes three dimensions: “Emotion exhaustion,” “depersonalization,” “diminished personal accomplishment,” with a total of 15 questions. The scale was scored using a 7-point Likert scale (1 = “completely inconsistent,” 7 = “completely consistent”). Among them, the sense of achievement reduction dimension is scored in all directions, and the score of each dimension is between 5 and 35 points. The higher the score, the more serious the Job Burnout. In this study, the Cronbach alpha coefficient of the scale was 0.874.

### 2.3 Data analysis

Descriptive statistical analysis and mediation analysis were performed in this study using SPSS 27.0 software, and all network analyses were performed using R (version 4.2.1). Unlike traditional methods (e.g., regression) that test linear relationships between variables, network analysis models constructs as ‘nodes’ (e.g., family support, cognitive reappraisal) and their associations as ‘edges’ (e.g., positive/negative correlations). This allows us to visualize which nodes are most influential (e.g., ‘core hubs’) and how effects spread across the system. First, the perceived social support – emotion regulation – job burnout network was constructed and visualized using the EBICglasso function from the R “qgraph” package, and the data were fitted with the Gaussian graphical model (GGM) (Epskamp et al., 2018), in which the total scores of the eight dimensions from the three scales were used as the eight nodes in the network, and the connecting lines between the nodes were regarded as the “edges,” and the thickness of the edges indicated the correlation between the nodes, with the blue edges indicating the positive correlation and the red edges indicating the negative correlation, and the matrix of the non-parametric Pearson partial correlation coefficient coefficients between the items was used as data input of the GGM. data input, and the optimal regularization parameters are determined by the graphical LASSO algorithm and the extended Bayesian information criterion to ensure the stability and interpretability of the GGM network structure (Foygel and Drton, 2010).

Bridge expected influence (BEI) was used as a key metric in this study to identify and quantify the role of bridge nodes connecting different psychological constructs within the network. BEI measures the total strength of a node’s direct connections to nodes in other communities, thereby reflecting its potential to facilitate or inhibit the transmission of effects across distinct functional clusters (e.g., between perceived social support and job burnout dimensions). A higher absolute BEI value indicates that the node plays a more substantial bridging role, either as a active propagator of positive influence or as a critical pathway for risk propagation.

#### 2.3.1 Centrality and stability

The community division matches exactly the “dimensional design objectives” of the scale employed, ensuring that nodes within each community serve the same measurement purpose and avoid confusion across community dimensions. Therefore, this study pre-defined the eight dimensional nodes from the Perceived Social Support, Emotion Regulation, and Job Burnout scales into three communities as follows: the PSSS community (Family Support, Friends Support, Other Support), the ERQ community (Cognitive Reappraisal, Expression Suppression), and the Job Burnout community (Emotional Exhaustion, Depersonalization, Diminished Personal Accomplishment). With the help of “bootnet” in R package to measure the centrality index, we select Betweenness, Closeness, Strength and Expected Influence as the core dimensions to identify the key elements affecting the network structure, and to deeply explore the indicators that have a core driving effect on perceived social support, emotion regulation and Job Burnout of primary and secondary school teachers. Additionally, the one-step bridge expected influence (BEI) method (Jones et al., 2019) was employed to evaluate bridge nodes across communities. This metric quantifies the total strength of a node’s direct connections to all nodes outside its own community—specifically, considering only direct one-step connections spanning the PSSS, ERQ, and Job Burnout communities. The computation was implemented using the bridge function from the R package “networktools,” with the aim of identifying core bridge nodes that connect the perceived social support, emotion regulation, and job burnout systems, and further analysing their mediating roles in the association between perceived social support and job burnout. Node predictability was calculated using the R package “mgm,” which quantifies the mutual explanatory capacity among nodes within the network structure (Haslbeck and Waldorp, 2017).

#### 2.3.2 Stability and accuracy of network structure

In order to verify the stability and accuracy of the whole network structure, this study examines the stability of the centrality index and the accuracy of the edges. Among them, the stability of the network nodes is assessed by a case-drop bootstrap test with 5,000 bootstraps and quantified by the correlation stability coefficient (CS coefficient). The results indicate that the correlation stability coefficient (CS-coefficient) should not fall below 0.25, and it is preferable to exceed 0.5 (Epskamp et al., 2018). The accuracy of edges is determined using a nonparametric bootstrap test to determine the 95% confidence interval of the edge (EDGE) estimated by 5,000 bootstraps, with smaller interval overlap indicating higher accuracy. In addition, the variability of nodes and edges is also analyzed by a nonparametric bootstrap test, and 5,000 bootstraps are performed to ensure the reliability of the results. In practical research, a CS value ≥0.50 is regarded as the ideal standard, while a CS value ≥0.25 represents the minimum acceptable threshold. This metric helps researchers evaluate the robustness of network inferences. Especially when designing interventions targeting central nodes, a higher CS value enhances the credibility and replicability of practical strategies.

#### 2.3.3 Network comparison

Previous studies have suggested that gender may influence individuals’ patterns of perceived social support and emotion regulation strategies (Okumura et al., 2022; van Middendorp et al., 2005). Among males, family support is a stronger predictor than support from friends or other significant others, whereas among females, both family support and support from significant others have the greatest impact. In addition, there are significant differences in the work stressors faced by elementary and secondary school teachers: elementary school teachers focus more on daily behavior management and basic competence development, while secondary school teachers need to cope with academic promotion pressure, the management of adolescent students, and more complex teaching tasks.

Traditional differences tests (e.g., *t*-test, ANOVA) can only compare the “mean differences” of variables (e.g., whether the mean burnout is different between men and women) and cannot answer the core question of this study: “Is there a difference in the pattern of variable association between different groups.” Therefore, in order to test the differences between primary and secondary school teachers by gender and by school year, we used the R package Network Comparison Test (NCT) (van Borkulo et al., 2023) for further analysis. Specifically, it includes network structure invariance test (i.e., the difference between the maximum edge strengths of the network), global strength invariance test (i.e., the difference between the sum of edge strengths), and edge strength invariance test (i.e., the difference between certain edges in the network).

## 3 Results

### 3.1 Common method bias test and descriptive statistics

The Harman one-way test (Dandan and Zhonglin, 2020) was used to examine the common method bias of the research data, and the results showed that the variance explained by the first factor was 26.731%,which was smaller than the critical criterion of 40%, indicating that there was no serious common method bias in this study.

Table 1 demonstrates the mean, standard deviation, skewness and kurtosis of the variables for the 351 participants grouped by gender. Judging from the skewness and kurtosis indicators, The absolute values of skewness were all less than 3, and the absolute values of kurtosis were all below 10; therefore, the data can be considered to approximate a normal distribution (Kim, 2013).

**Table 1:** Descriptive statistics of each variable.

Variables	Male (*N* = 107)	Female (*N* = 244)	*t*	Skewness	Kurtosis	Cronbach’s alpha
Age	38.98 ± 7.731	37.33 ± 8.166		0.257	−0.648	
Perceived Social Support	56.47 ± 13.165	60.02 ± 13.018	−2.338*	−0.551	0.351	0.943
Emotion regulation	41.70 ± 13.675	41.45 ± 11.957	0.166	−0.370	−0.654	0.899
Job Burnout	31.33 ± 15.802	29.68 ± 17.348	0.870	0.471	−0.103	0.874

### 3.2 Correlation analysis

**Figure 1:**
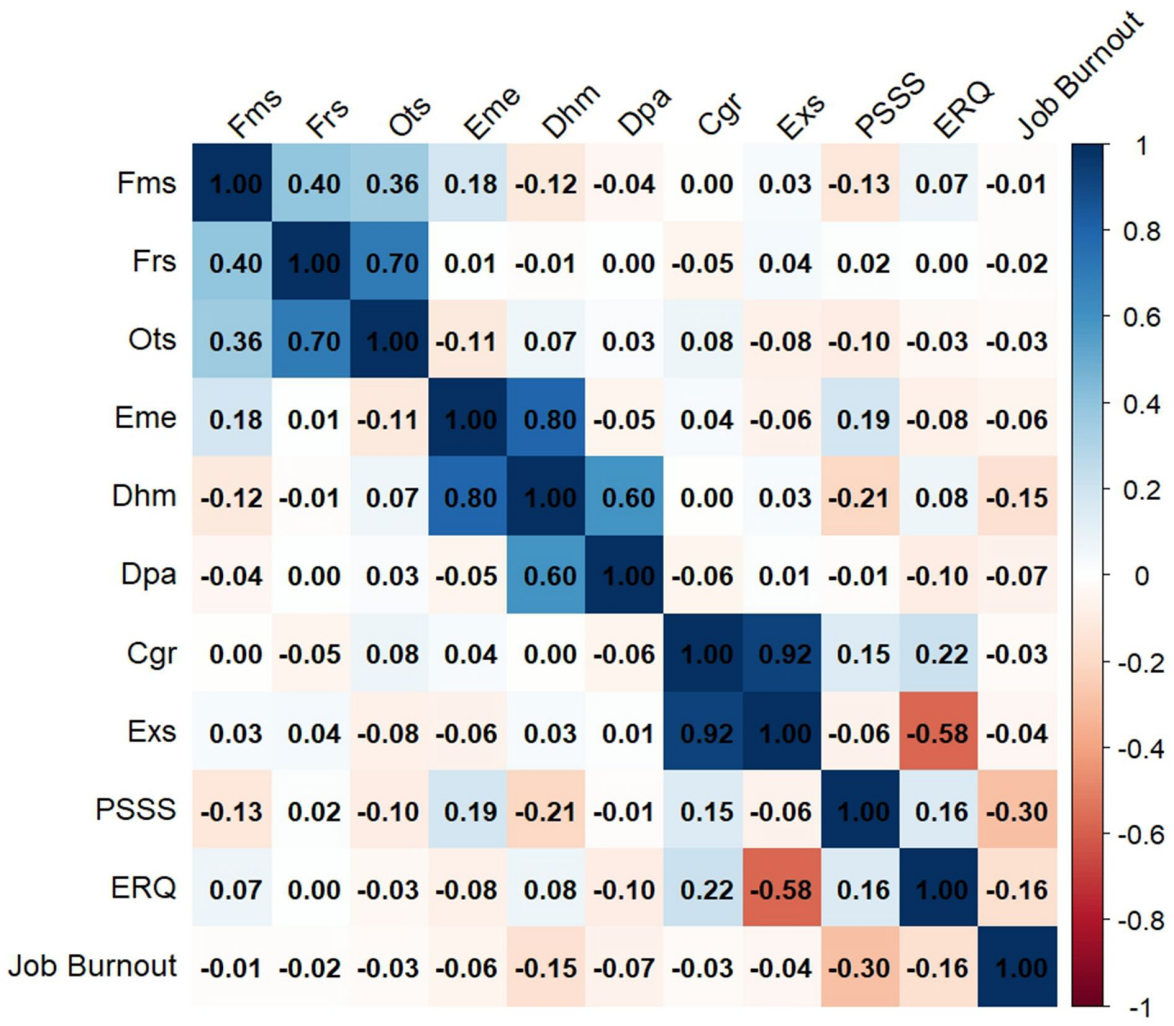
Partial correlation coefficients between different nodes. Fms, family support; Frs, friends support; Ots, other support; Eme, emotion exhaustion; Dhm, dehumanization; Dpa, diminished personal accomplishment; Cgr, cognitive reappraisal; Exs, expression suppression; PSSS, perceived social support; ERQ, emotion regulation questionnaire.

Figure 1 displays the partial correlation coefficients among different variables used to examine the associations between them. The results of the analysis showed that perceived social support was positively correlated with emotion regulation (*r* = 0.16) and negatively correlated with job burnout (*r* = −0.30). In addition, emotion regulation was also negatively correlated with Job Burnout (*r* = −0.16). Between different dimensions, family support had the strongest relationship with emotion exhaustion (*r* = 0.18), Within the same dimension, the correlation between emotional exhaustion and depersonalization is the strongest (*r* = 0.80).

### 3.3 Network analysis

**Figure 2:**
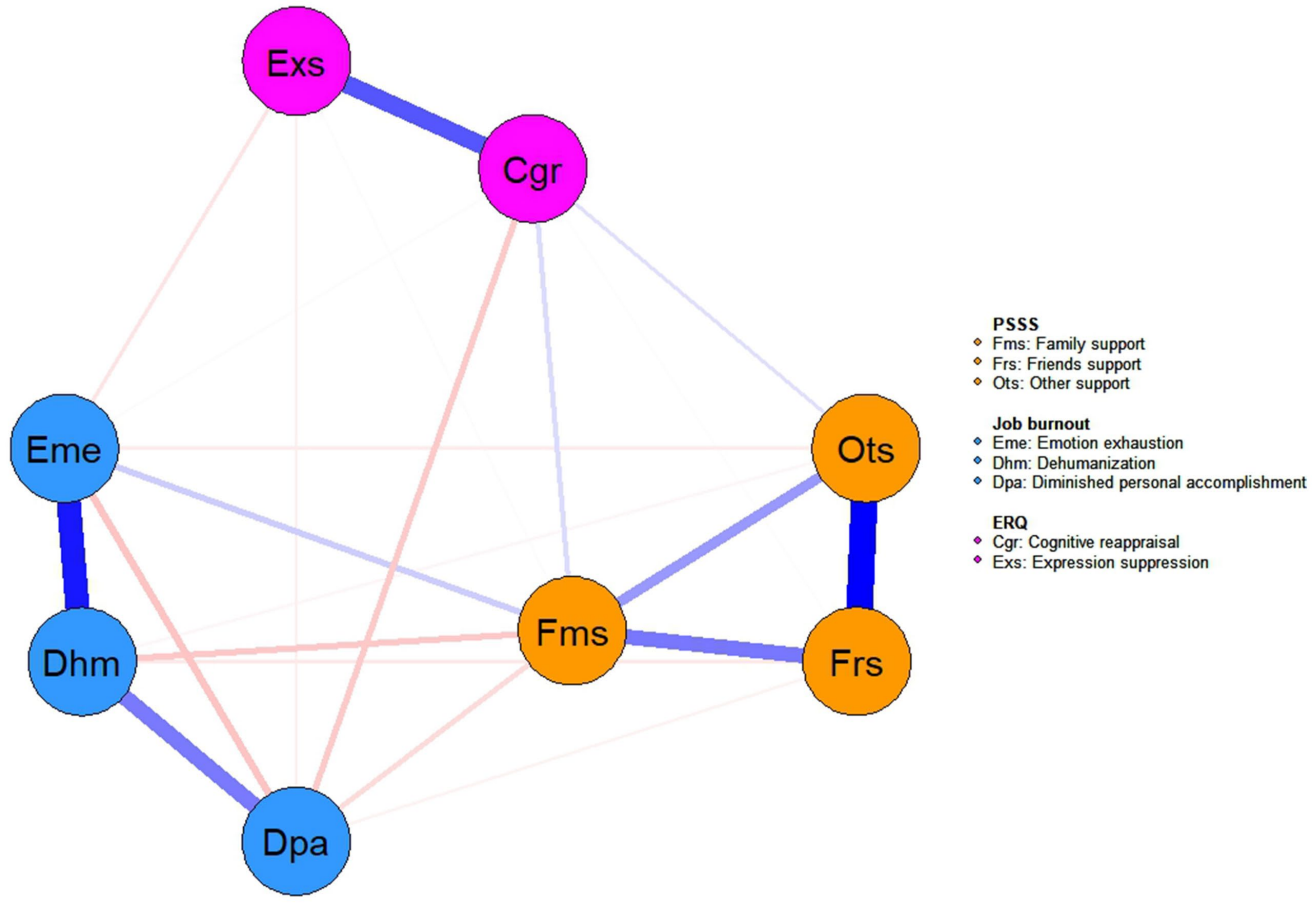
Network structure of perceived social support, emotional regulation and job burnout.

We constructed a “perceived social support-emotion regulation-job burnout” network with 8 nodes and up to 28 possible links. The results show that there are 22 edges in the network, accounting for 78.57% of all possible edges, with an average weight of 0.072. The analysis showed that the strongest association was between “Other support” and “Friends support” in terms of perceived social support, while the strongest association was between “emotional exhaustion” and “depersonalization” in terms of job burnout. In addition, the core cross-community associations were “family support” and “emotional exhaustion.” See Figure 2 for details, and Supplementary Table S1 of non-zero edge weights for cross-dimension connections.

### 3.4 Node centrality and bridge symptoms

**Figure 3:**
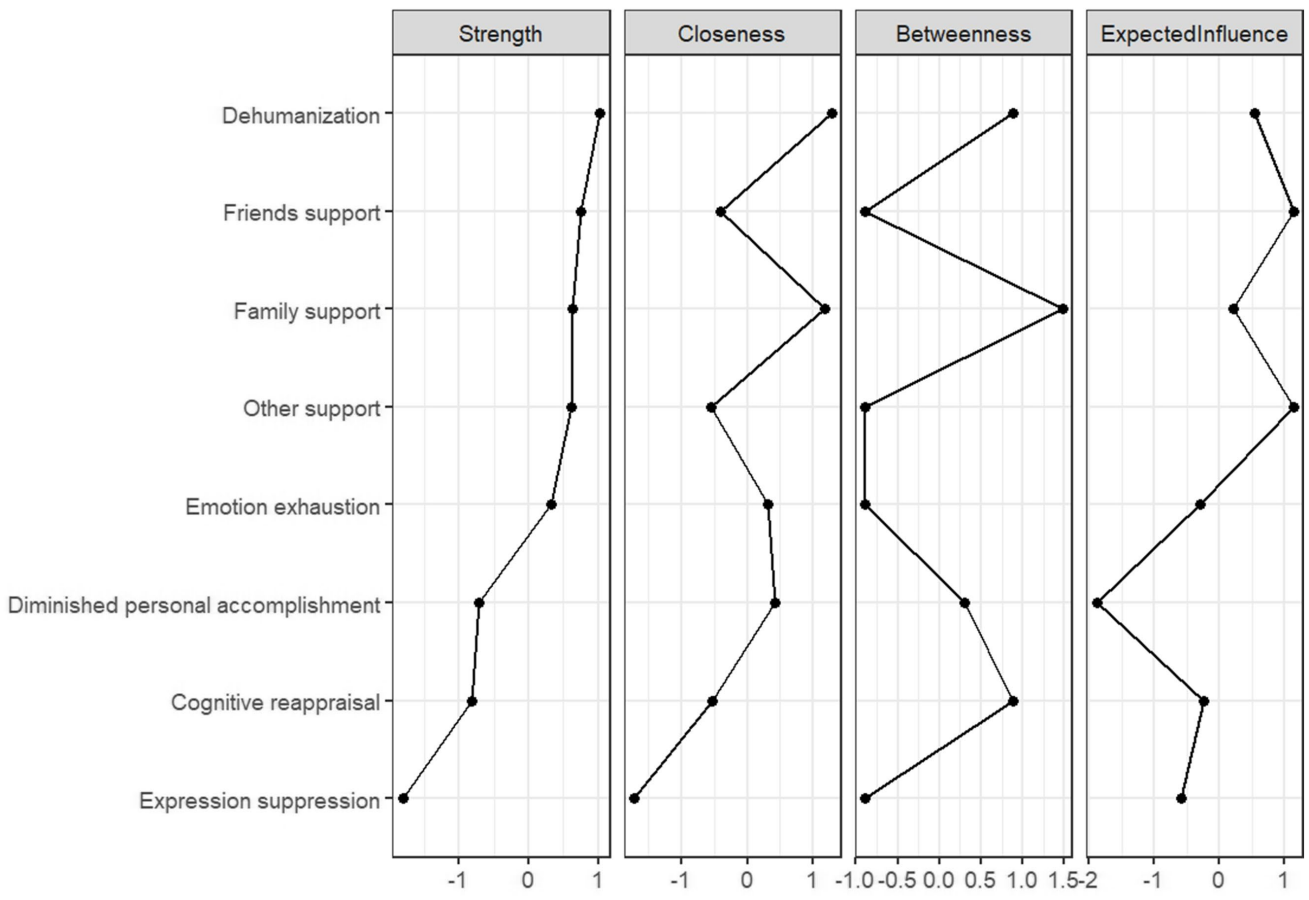
Comparison of strength, proximity, mediation, and expected impact of different nodes.

Figure 3 and Supplementary Table S2 shows the data values of each network node in terms of strength, proximity, mediation and expected impact. In the perceived social support network, the node representing family support demonstrated higher betweenness centrality and closeness centrality compared to other nodes, which indicates that it is more strongly connected to the other nodes in the network and that many of the shortest paths between nodes feed through it. In the network of nodes for job burnout, the depersonalization node shows stronger proximity and expected impact. In the emotion regulation network, the cognitive reappraisal node exhibited higher values than the expression suppression node across all centrality measures.

**Figure 4:**
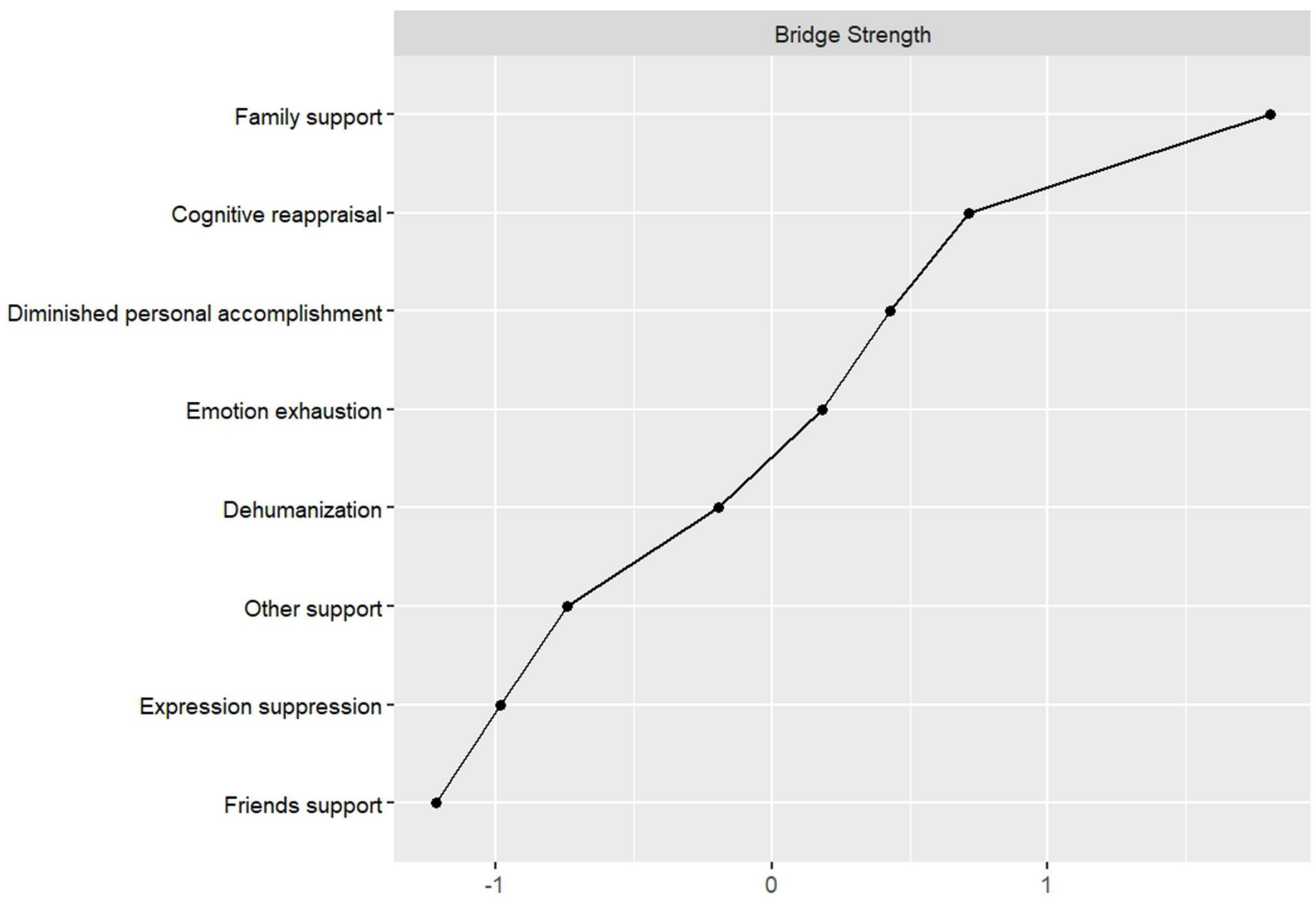
Bridging strength of individual nodes.

Figure 4 displays the bridge strength values of nodes across the three networks: family support demonstrated the highest node strength within the perceived social support network, reduced personal accomplishment exhibited the strongest node strength in the job burnout network, and cognitive reappraisal showed the greatest node strength in the emotion regulation network. Throughout the network family support had the highest Bridge Strength, meaning it acts as a ‘bridge’ between social support and burnout—this aligns with COR Theory, as family support may replenish emotional resources that directly reduce depersonalization.

### 3.5 Network stability and accuracy

**Figure 5:**
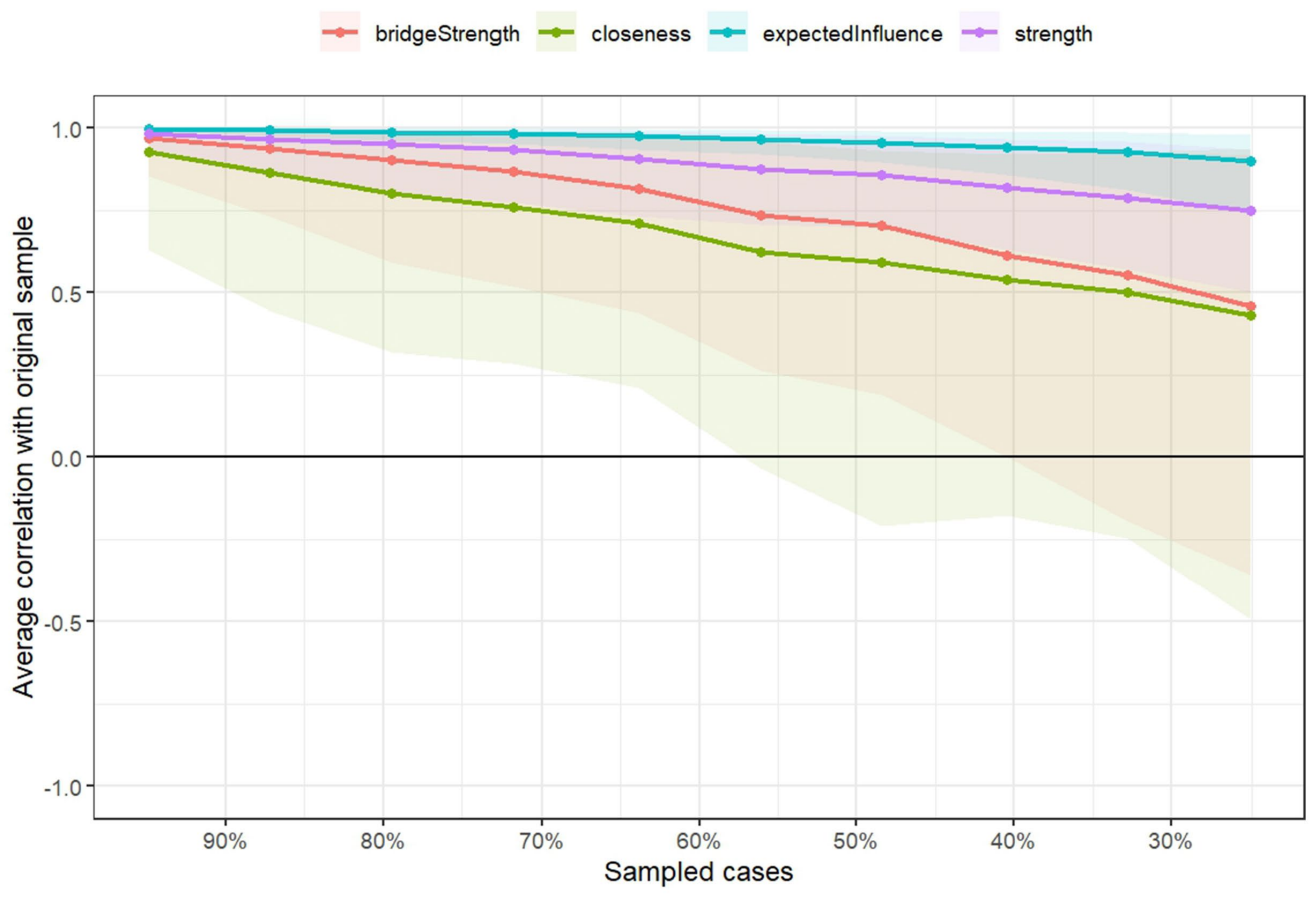
Correlation between node centrality indicators and original samples under different sampling ratios.

Figure 5 presents the network stability analysis results, obtained through a case-dropping bootstrap procedure. The correlation stability coefficient (CS-C) was 0.749 for edges and expected influence, and 0.439 for strength centrality. These results indicate that the core framework of the network—particularly edges and expected influence—exhibits good stability. Even after removing up to 75% of the sample, the main conclusions remain robust. In terms of network accuracy, the narrow 95% confidence intervals derived from edge bootstrap suggest minimal estimation error in edge weights, supporting the high reliability of the measurements. In addition, the bootstrap difference test shows that most two-by-two comparisons of expected influence and node strength are statistically significant (the specific differences can be referred to Supplementary Figures S2, S3), which further verifies the reliability of the differences in the core indicators.

### 3.6 Network comparison

**Figure 6:**
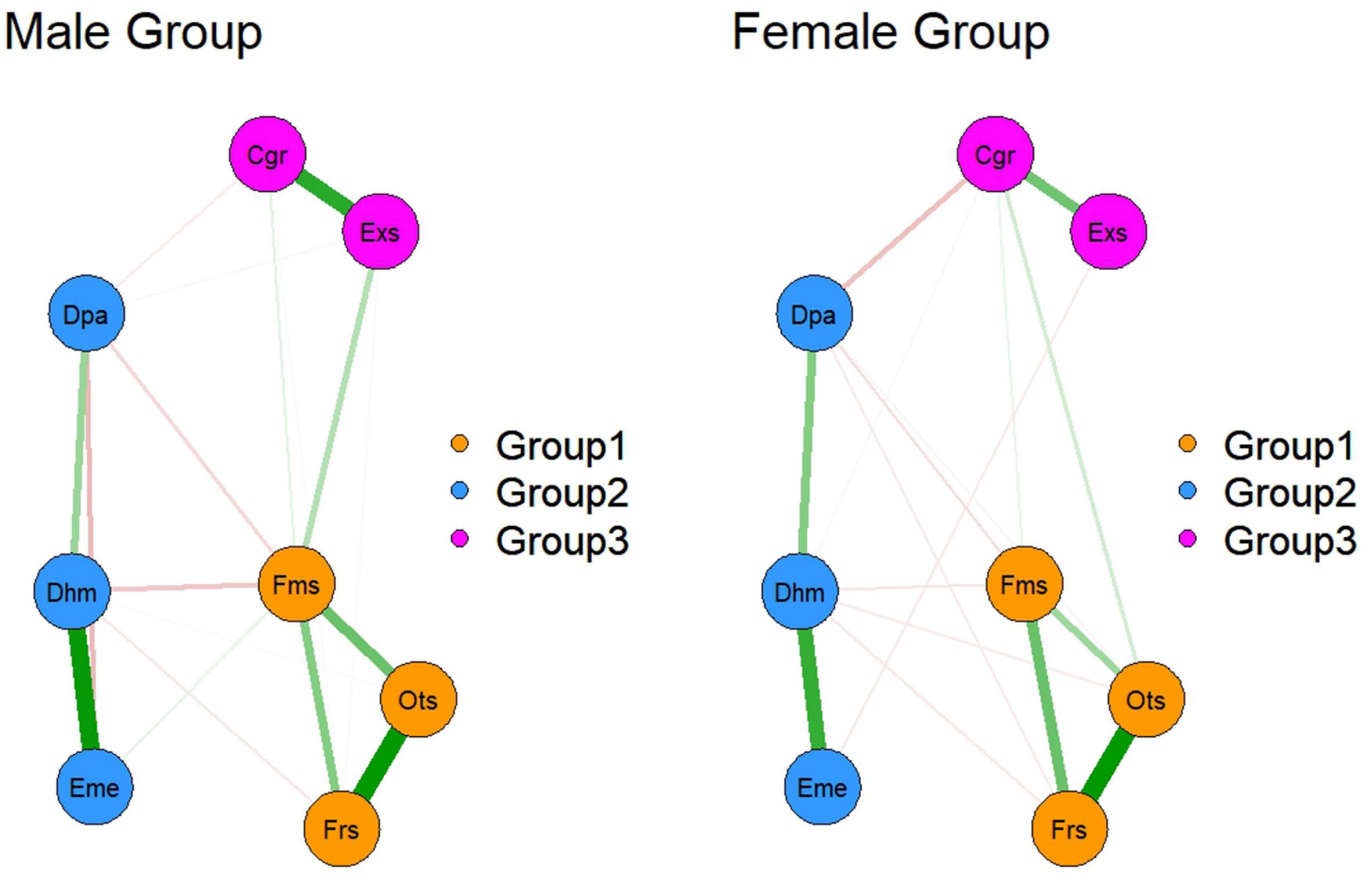
Comparison of network structure by gender. Group 1, perceived social support; Group 2, job burnout; Group 3, emotion regulation questionnaire.

The results of the NCT test showed that no significant gender differences were found in terms of network global strength (male: 2.27 vs. female: 1.77, *S* = 0.50, *p* = 0.14), network structure (*M* = 0.17, *p* = 0.74), and individual edge weights (all *p*-values >0.05 after Holm-Bonferroni correction). The sample sizes for gender subgroups were *N* = 107 (male) and *N* = 244 (female). In addition, no significant differences were found between the elementary and secondary teacher groups in terms of network global strength (1.84 vs. 2.07, *S* = 0.23, *p* = 0.40) and network structure (*M* = 0.21, *p* = 0.35). The sample sizes for school level subgroups were *N* = 178 (elementary) and *N* = 173 (secondary). All networks were estimated separately with identical nodes (8 nodes representing dimensions of perceived social support, emotion regulation, and Job burnout) and consistent tuning parameters: the gamma parameter for EBICglasso regularization was set to 0.05 for all subgroup networks. Together, these results suggest that the measured network characteristics do not differ significantly between genders and instructional sections, and Figure 6 illustrates the network structure seen by gender. Network structure by instructional section See Supplementary Figure S4 for details.

### 3.7 Mediating effects analysis

In order to further explore the relationship between the variables, the study used Model 4 in the Process 4.1 macro plug-in developed by Hayes for mediating effects analysis (Preacher and Hayes, 2008). In the study, perceived social support was used as the independent variable, job burnout as the dependent variable, and emotion regulation as the mediating variable. Also, in order to control the effect of latent variables, the study included age and gender as control variables in the model. Before model analysis, all variables were standardized to improve the explanatory power of the model. To ensure the robustness and precision of the analysis results, the samples were subjected to 5,000 Bootstrap replications and a standard 95% confidence interval was set.

**Figure 7:**
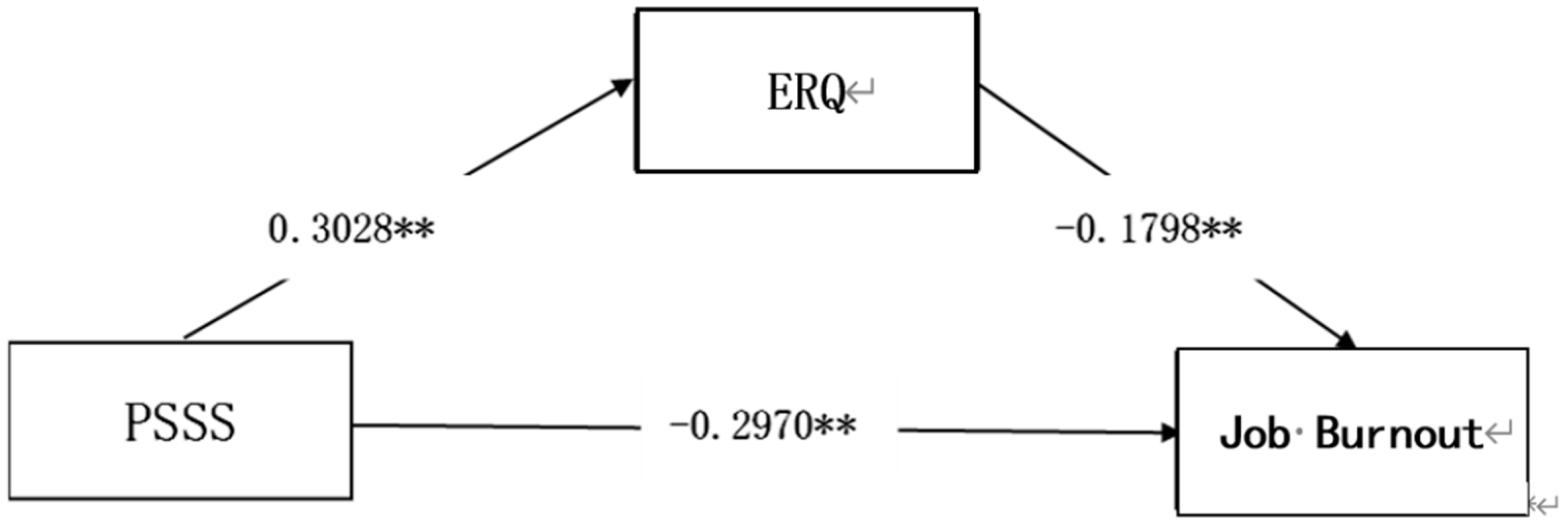
Model diagram of mediating effect. PSSS, perceived social support; ERQ, emotion regulation questionnaire.

The analysis results indicated (Table 2) that emotion regulation served as a significant mediator in the relationship between perceived social support and job burnout among elementary and secondary school teachers, with an effect value of −0.0544 and 95%CI (−0.0937, −0.0197). Specifically, perceived social support was positively associated with emotion regulation (*β* = 0.3028, *p* < 0.01), and emotion regulation was negatively associated with job burnout (*β* = −0.1798, *p* < 0.01), which is consistent with the hypothesis that emotion regulation statistically mediates the association between perceived social support and job burnout. Figure 7 visualizes this mediating effect.

**Table 2:** Mediating effect test.

Path	Effect value	*p*-value	LLCI	ULCI	Effect size
Total effect	−0.3514	<0.01	−0.5795	−0.3233	100%
Direct effect	−0.2970	<0.01	−0.5137	−0.2492	84.52%
Intermediary effect	−0.0544		−0.0937	−0.0197	15.48%

## 4 Discussion

This study employs network analysis to examine the structural relationships among perceived social support, emotion regulation, and job burnout in primary and secondary school teachers, with a specific focus on the mediating role of emotion regulation between perceived social support and job burnout. The present study attempts to provide a deeper, or at least different, understanding of Job Burnout and its links to perceived social support and emotion regulation. New psychological approaches such as network and mediation modeling may provide new perspectives for describing, conceptualizing, understanding, and dealing with job burnout.

### 4.1 Perceived social support, emotion regulation, and job burnout cross-sectional network analysis

This study constructed a network structure to examine the associations among perceived social support, emotion regulation, and job burnout at the symptom level among elementary and secondary school teachers. The strongest connections were observed within each variable, while cross-variable connections were relatively weak—a pattern consistent with previous research (Xu et al., 2025) and the Conservation of Resources Theory (COR theory), which posits that individuals under stress tend to mobilize similar types of resources to cope with domain-specific demands. Notably, the three strongest cross-variable connections identified were between “family support” and “emotional exhaustion,” “family support” and “depersonalization,” as well as “cognitive reappraisal” and “reduced personal accomplishment.” Although inter-variable links were generally weak, these specific associations suggest meaningful pathways through which social support and emotion regulation may influence distinct dimensions of job burnout, highlighting potential targets for mechanistic research and intervention strategies.

In this study of perceived social support as an external factor affecting job burnout, we observed the results that family support was positively associated with emotional exhaustion and negatively associated with depersonalization and reduced personal accomplishment, revealing the differential impact of family support on different dimensions of job burnout in a group of elementary and secondary school teachers (Greenglass et al., 1996). Teachers need to continuously perform “emotional labor” in their work, and family support, if accompanied by additional emotional needs, will lead to the continuous depletion of emotional resources, forming a positive path of “family interaction → emotional depletion” (Huang et al., 2023). This is consistent with the extended perspective of Conservation of Resources Theory (COR), which suggests that when external support requires more emotional input than it replenishes, the support itself may become a source of stress (Chen et al., 2024). At the core of depersonalization is “emotional detachment,” i.e., showing indifference and dehumanizing perceptions of the person with whom one is working. However, the emotional connection provided by family support, such as the understanding and close interaction of family members, can maintain teachers’ “emotional sensitivity” and thus reduce their “defensive indifference” due to stress in the workplace (Varela et al., 2023). This supports the role of family support as a cross-community bridge that directly counteracts depersonalization. The “value affirmation” implicit in family support (e.g., “your work is meaningful”) buffers teachers from self-doubt due to work-related frustrations (e.g., student management difficulties, evaluative pressures) and maintains their identification with professional values, thereby reducing the risk of diminished personal fulfillment. of risk. This mechanism is in line with the “social identity theory” – the positive feedback from the family as a core reference group reinforces teachers’ professional identity (Huang et al., 2024). In addition, this study found that family support is the core dimension in alleviating teacher Job Burnout, which contrasts with the findings of another study which suggested that it is friend and colleague support that is the main factor in reducing job burnout (Rogers et al., 2016), this discrepancy may reflect population characteristics or contextual specificities, suggesting that the effect of perceived social support may depend on the alignment among support type, job burnout dimension, and group attributes—a finding consistent with research among primary care workers, where work-family support played a major role in reducing job burnout (Liu et al., 2025). In addition, in the estimated psychological network, the most central nodes by strength were depersonalization, friend support and family support, and the most influential nodes in terms of expected impact were other support, friend support and depersonalization. Both indicators emphasize the central role of depersonalization.

In addition, a significant negative correlation was found between cognitive reappraisal and diminished sense of personal accomplishment, a result that provides key evidence for understanding the protective mechanisms of emotion regulation strategies against teacher job burnout. Cognitive reappraisal, as an active emotion regulation strategy, can directly reduce the impact of negative emotions on the sense of professional worthiness by changing the cognitive interpretation of stressful events (Donker et al., 2020). From the perspective of resource conservation theory, cognitive reappraisal reduces the rate of depletion of emotional resources by optimizing the cognitive processing of work stress, making it easier for teachers to perceive their self-worth in their teaching outcomes, and thus slowing down the decline in their sense of personal accomplishment (Liu and Qin, 2005). While previous studies have established that teachers proficient in emotion regulation experience reduced emotional exhaustion and higher job satisfaction (Chang, 2020), and that cognitive reappraisal promotes positive reinterpretation of workplace challenges (Zeng et al., 2024), this study specifically highlights its function in countering the loss of personal accomplishment. Rather than merely summarizing known effects, these findings refine the theoretical mechanism by positioning cognitive reappraisal as a resource-preserving cognitive tool that directly targets the erosion of professional meaning—a dimension particularly salient in educational settings.

Bridging Expected Impact (BEI) is used to measure the degree of interconnectedness between communities, with higher values indicating a greater likelihood of activating other communities (Chen et al., 2022). Family support had the highest and positive BEI and was the core cross-system bridge, which is consistent with previous research findings (Putri et al., 2023), indicating that family support is not only a resource within the perceived social support system, but is also deeply linked to the emotion regulation and Job Burnout systems, and can be utilized to buffer teachers’ emotional exhaustion and improve job burnout. Through the Bridge Expected Influence (BEI) analysis, it was observed that friend support consistently exhibited the lowest BEI values among all nodes within the perceived social support community. Previous research has shown that nodes with higher negative BEI can be considered as protective factors (Liu et al., 2023). This suggests to us that friend support might be more effective as a protective factor to reduce job burnout among primary and secondary school teachers, and this finding has important clinical reference value. Family support and friend support form a complementary pattern of “active regulation” and “passive buffering” in the network: family support promotes the overall optimization of the job burnout network through high-intensity cross-system connections, while friend support maintains the network through stable local protective effects. This suggests that practical interventions should leverage the strengths of both through stratified strategies: for teachers already exhibiting significant job burnout symptoms, prioritizing the enhancement of family support to rapidly restructure their psychological network; for preventive populations, focusing on cultivating supportive friend networks to strengthen overall resilience. Such role-targeted interventions are expected to improve both the efficiency and sustainability of mental health support for teachers.

### 4.2 Mediation analysis of emotion regulation in comprehending the impact of perceived social support on job burnout

We further constructed a model with emotion regulation as a mediating variable. The results of the model analysis show that perceived social support can directly predict job burnout and also indirectly affect job burnout through the mediating variable of emotion regulation. Emotion regulation plays a bridging role between perceived social support and job burnout, this is consistent with the chain pattern of “perceived social support → emotion regulation → job burnout” (Kahn et al., 2006). Perceived social support, as an external resource, first affects an individual’s ability to regulate emotions, i.e., the more adequate the social support perceived by a teacher, the more likely he or she is to develop positive emotion-regulation strategies, e.g., Cognitive reappraisal (Li et al., 2024). Effective emotion regulation can directly mitigate the negative effects of job stress on job burnout and reduce symptoms such as emotional exhaustion and depersonalization (Cadariu and Rad, 2025). This is consistent with the cross-system connectivity characterizing the emotion regulation nodes in the network analysis, suggesting that emotion regulation does not work in isolation, but is a key mediating hub between perceived social support and job burnout. This mechanism reveals a dual pathway through which perceived social support influences job burnout: both by directly providing emotional comfort or practical help to buffer stress, and by indirectly blocking the development of job burnout through the enhancement of emotion regulation. This synergy of external support and internal regulation provides a more systematic perspective on the maintenance of teachers’ mental health, and points to the direction of intervention practice – the need to simultaneously strengthen the perceived social support system and the ability to regulate emotions, in order to form a more comprehensive The study also points to the direction of intervention practice – the need to strengthen the perceived social support system and emotional regulation ability simultaneously to form a more comprehensive protective barrier.

### 4.3 Limitations

There are some limitations to this study. First, the study relied only on self-reported data, which may be affected by social expectation bias or immediate emotional state, resulting in limited objectivity of the results. Second, due to the cross-sectional research design, only the co-occurring associations between variables could be revealed, and the causal time sequence between variables could not be clarified. Third, despite the inclusion of primary and secondary school teachers, groups such as special education teachers and vocational college teachers were not covered, and the effects of segmentation characteristics such as teaching age and teaching subjects were not fully considered. Fourth, although the mediating role of emotion regulation was verified, the association between perceived social support and job burnout may involve more potential mediating variables (e.g., teaching efficacy, work engagement), and the explanation of the association mechanism is not comprehensive enough. Given that Harman’s single-factor test cannot serve as a definitive examination for common method bias, and considering that more rigorous approaches—such as confirmatory factor analysis with a common latent factor or the marker variable technique—were not employed in this study, we acknowledge certain limitations in controlling for common method variance. Future research should incorporate multiple validation strategies, such as introducing marker variables or performing confirmatory factor analysis, to more comprehensively address this bias. Furthermore, since only two schools were included in this study, we were unable to reliably estimate or control for potential school-level confounding variables, which may simultaneously influence teachers’ perceived social support, emotion regulation strategies, and job burnout levels. Subsequent studies should recruit teacher samples from a larger number of schools to mitigate the impact of such confounding factors. Since this study is based on Chinese cultural background and does not fully explore the potential impact of cultural traits on variable relationships, subsequent studies should clearly include cultural dimension analysis, or verify the moderating effect of cultural context on variable relationships through cross-cultural comparative research.

## 5 Conclusion

Job burnout is a prevalent psychological issue among primary and secondary school teachers, posing considerable threats to their mental well-being. To systematically identify relevant risk and protective factors, this study employed network analysis and mediation modeling to examine the structural relationships among perceived social support, emotion regulation, and job burnout within the teacher population. The findings reveal a protective network mechanism centered on the “social support–emotion regulation” pathway, highlighting the critical roles of family support and cognitive reappraisal in mitigating job burnout. These results provide a theoretical foundation and specific targets for implementing precise mental health interventions—such as improving the quality of family support and enhancing cognitive reappraisal skills.

## Data Availability

The original contributions presented in the study are included in the article/Supplementary material, further inquiries can be directed to the corresponding authors.
