# Supplementary material for: A network analysis of the relationship between perceived social support, emotion regulation, and job burnout among elementary and middle school teachers
Source: Front Psychol. 2025 Oct 31;16:1704862. doi: 10.3389/fpsyg.2025.1704862 (PMC12615160; doi:10.3389/fpsyg.2025.1704862)
Supplement: Supplementary file 1 [file Table_1.DOCX]

Table S1: Table of non-zero edge weights for cross-dimension connections

| Node 1 | Node 2 | Estimate |
| --- | --- | --- |
| Friends support | Other support | 0.612 |
| Emotion exhaustion | Dehumanization | 0.554 |
| Cognitive reappraisal | Expression suppression | 0.405 |
| Family support | Friends support | 0.323 |
| Dehumanization | Diminished personal accomplishment | 0.320 |
| Family support | Other support | 0.245 |
| Emotion exhaustion | Diminished personal accomplishment | -0.138 |
| Family support | Dehumanization | -0.130 |
| Cognitive reappraisal | Diminished personal accomplishment | -0.128 |
| Family support | Emotion exhaustion | 0.121 |
| Expression suppression | Emotion exhaustion | -0.064 |
| Friends support | Dehumanization | -0.052 |
| Other support | Emotion exhaustion | -0.046 |
| Expression suppression | Diminished personal accomplishment | -0.035 |
| Family support | Cognitive reappraisal | 0.084 |
| Other support | Cognitive reappraisal | 0.073 |
| Friends support | Diminished personal accomplishment | -0.025 |
| Other support | Dehumanization | -0.019 |
| Cognitive reappraisal | Emotion exhaustion | -0.014 |
| Friends support | Cognitive reappraisal | 0.007 |
| Family support | Expression suppression | 0.012 |

Table S2: The data values of each network node in terms of strength, proximity, intermediation, and expected impact

| Node | Strength | Betweenness | Closeness | ExpectedInfluence |
| --- | --- | --- | --- | --- |
| Family support | 0.634 | 1.479 | 1.178 | 0.210 |
| Friends support | 0.747 | -0.887 | -0.401 | 1.141 |
| Other support | 0.623 | -0.887 | -0.549 | 1.139 |
| Emotion exhaustion | 0.325 | -0.887 | 0.318 | -0.293 |
| Dehumanization | 1.030 | 0.887 | 1.278 | 0.534 |
| Diminished personal accomplishment | -0.721 | 0.296 | 0.425 | -1.890 |
| Cognitive reappraisal | -0.825 | 0.887 | -0.530 | -0.247 |
| Expression suppression | -1.813 | -0.887 | -1.719 | -0.594 |


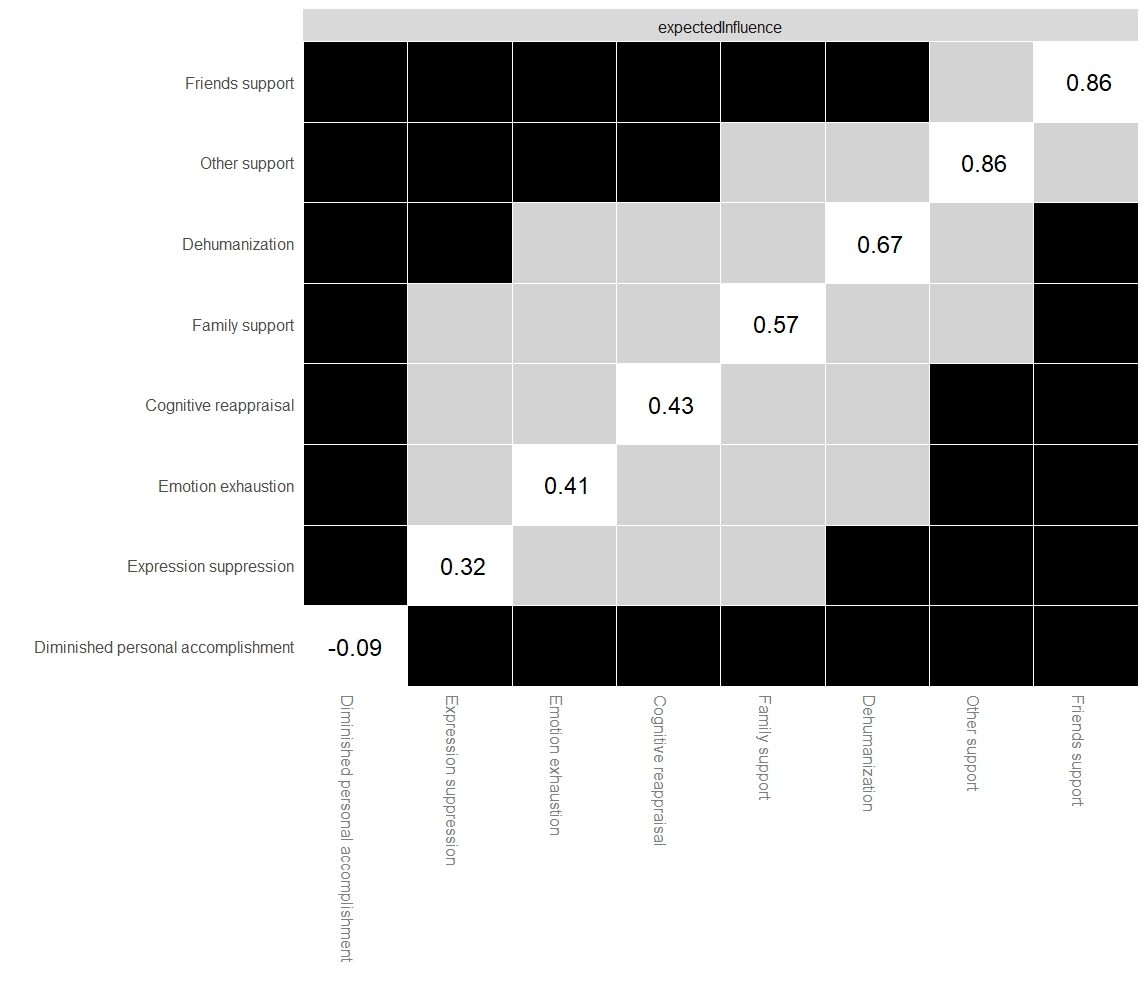


Figure S2: Bootstrap difference test for the expected impact of each node


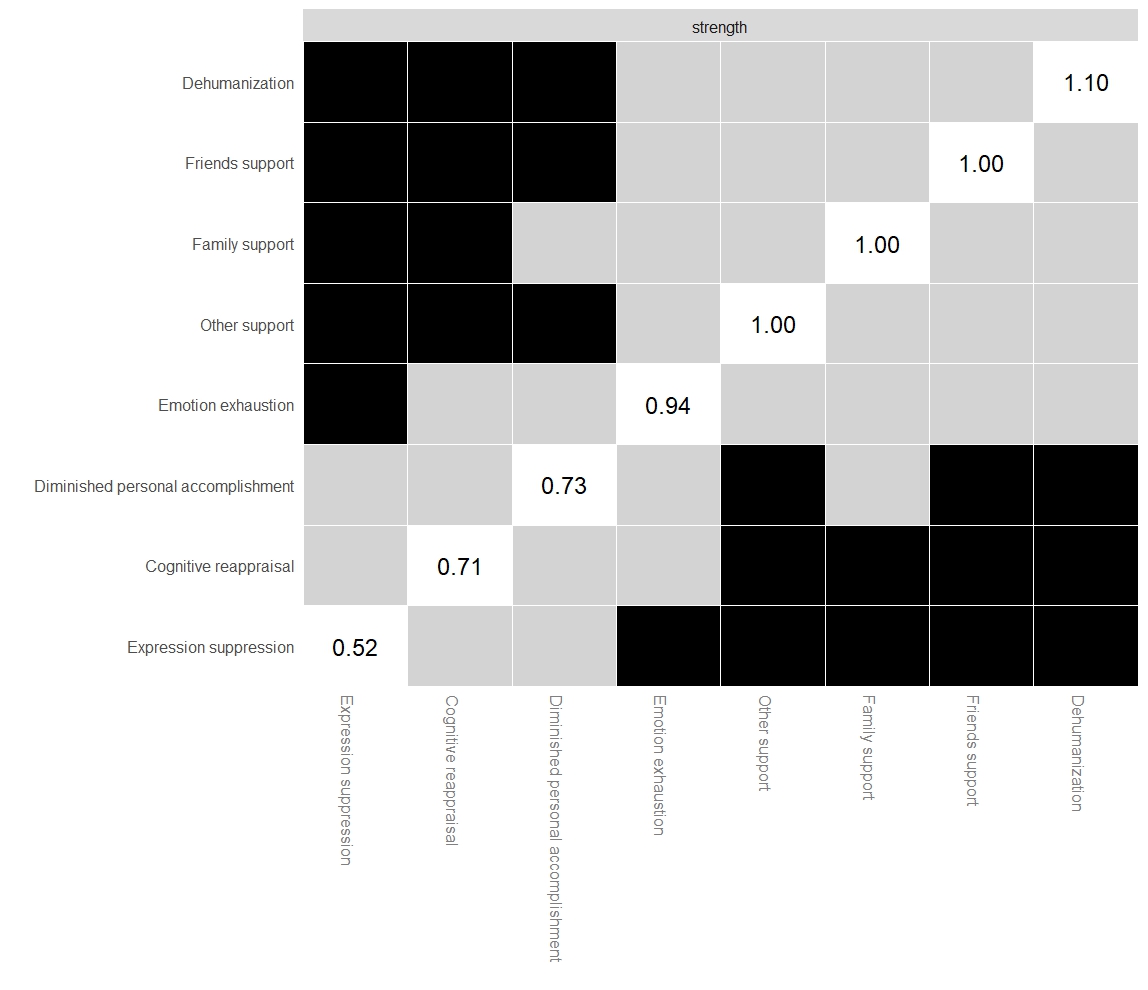


Figure S3: Bootstrap difference test between node intensities
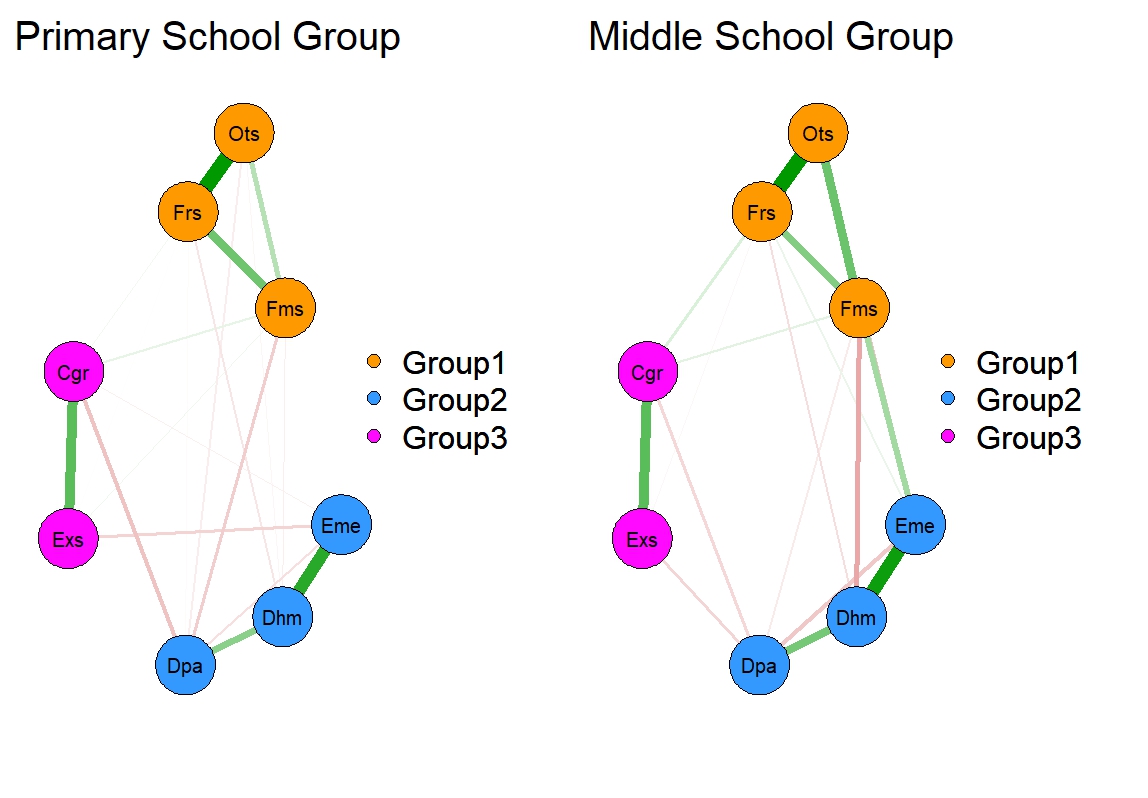


Note：Group1:Perceived Social Support; Group2:Job Burnout; Group3 :Emotion Regulation Ability

Figure S4: Network structure of different teaching sections
